# Operation of a Wind Turbine-Flywheel Energy Storage System under Conditions of Stochastic Change of Wind Energy

**DOI:** 10.1155/2014/643769

**Published:** 2014-08-18

**Authors:** Andrzej Tomczewski

**Affiliations:** Institute of Electrical Engineering and Industrial Electronics, Poznań University of Technology, Piotrowo 3A, 60-965 Poznań, Poland

## Abstract

The paper presents the issues of a wind turbine-flywheel energy storage system (WT-FESS) operation under real conditions. Stochastic changes of wind energy in time cause significant fluctuations of the system output power and as a result have a negative impact on the quality of the generated electrical energy. In the author's opinion it is possible to reduce the aforementioned effects by using an energy storage of an appropriate type and capacity. It was assumed that based on the technical parameters of a wind turbine-energy storage system and its geographical location one can determine the boundary capacity of the storage, which helps prevent power cuts to the grid at the assumed probability. Flywheel energy storage was selected due to its characteristics and technical parameters. The storage capacity was determined based on an empirical relationship using the results of the proposed statistical and energetic analysis of the measured wind velocity courses. A detailed algorithm of the WT-FESS with the power grid system was developed, eliminating short-term breaks in the turbine operation and periods when the wind turbine power was below the assumed level.

## 1. Introduction

Environmental issues included in long term power strategies of different countries and high accessibility of the renewable sources of energy and their significant potential are the main reasons for an increase in the share of renewable sources of energy in the global generation of electrical energy. When it comes to the widely available solar and wind energy, one should however pay attention to great fluctuations of the converters output power related to a stochastic nature of the irradiation changes *E*_*x*_ and wind velocity *v*_*w*_ in time. The instability has a negative impact on the cooperation of wind and solar sources with the power grid system [1–3]. The issue is important for systems with a high percent share of renewable sources of energy, particularly in those without output power stabilisation [2, 4]. The operation of unsustainable sources of energy can cause problems related to stabilisation of a section of a power grid system and generate additional costs related to maintaining the periodically activated conventional sources in a standby mode [5, 6].

Energy storage in industrial applications is a current issue and the research in the area led to some practical applications of batteries, artificial and natural compressed air energy storage (CAES), supercapacitors, superconducting magnetic energy storage (SMES), flywheel energy storage, and so forth, [4, 7–13]. Despite technical sophistication and high costs, their application area in high power systems is gradually extending. There are a growing number of technical devices including energy storage and further recovery as a part of normal operation, for example, emergency supply systems, pumped storage power plants, and hybrid and electrical cars. Due to growing significance of such kind of solutions used in economics of highly developed countries the problem should be considered with regard to widely understood optimization and economic aspects [11, 14].

An important issue of the practical application of wind sources is to mitigate the effects of output power fluctuations resulting from the stochastic nature of the wind energy changes in time, when working with a power grid system [15]. Long-lasting (e.g., for several hours) breaks in power generation in wind sources related to the decrease in the wind kinetic energy can be determined with the use of computer-assisted systems of the output power prediction [16–18]. The situation differs when the breaks are short (up to several minutes) and impossible to predict due to their generation mechanism. Moreover, the frequency and duration of short breaks depend on the parameters of the implemented wind turbine—mainly on its cut-in velocity *v*_cut-in_.

**Figure 1:**
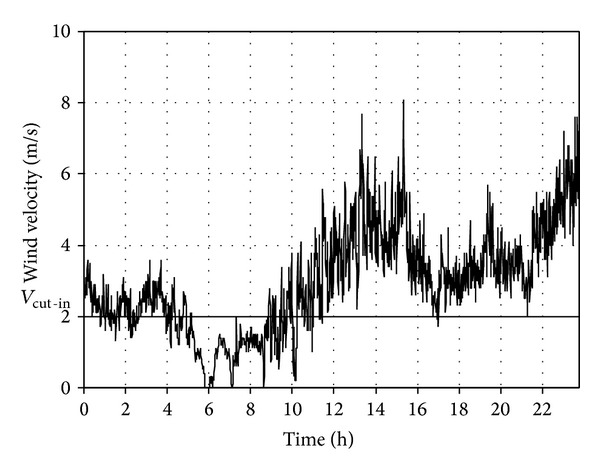
Circadian wind velocity changes recorded on 1 March 2008 in Strzyżów (South Eastern Poland) at the height of *h*_*p*_ = 10 m above the ground level.

**Figure 2:**
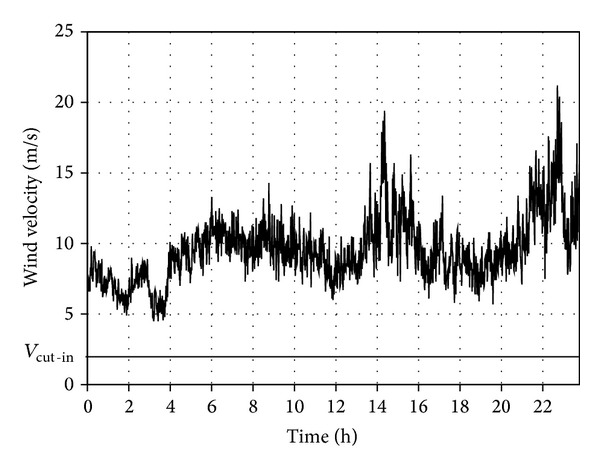
Circadian wind velocity changes recorded on 5 March 2008 in Strzyżów (South Eastern Poland) at the height of *h*_*p*_ = 10 m above the ground level.

Figures 1 and 2 present two circadian curves of wind velocity changes obtained by measurements. The measurements were made on 1 and 5 March 2008 in a meteorological station in Strzyżów (South Eastern Poland) at the height of *h*_*p*_ = 10 m above the ground level.

The obtained values were recalculated for the height of *h*_WT_ = 60 m above the land level, corresponding to the position of the wind wheel hub used for further analyses of Enercon E53 turbine, according to the following relationship:

(1) $$\begin{array}{c} v_{w\text{WT}}=v_{w}{\left(\frac{h_{\text{WT}}}{h_{p}}\right)}^{\alpha }, \end{array}$$

where *α* is the aerodynamic coefficient of terrain roughness, *h*_*p*_, *h*_WT_ are the heights of the wind velocity and wind wheel hub measurement, quoted in reference to the land level, *v*_*w*_ is the wind velocity at the measurement height *h*_*p*_, *v*_*w*WT_ is the recalculated wind velocity.

The model of wind velocity vertical profiles expressed in the formula (1) is simplified but sufficient for the purpose of the study.

The above-mentioned type of wind turbine with nominal power of *P*_WTN_ = 800 kW was used in all calculations and simulations done for the purpose of the study. The horizontal line marked in Figures 1 and 2 stands for the cut-in velocity, which for the reference type of turbine is *v*_cut-in_ = 2 m/s. The analysis of the curve presented in Figure 1 indicates an almost 5-hour break (long-lasting break) in power generation and many short turbine cut-outs from the power grid system. For the course presented in Figure 2 the average wind velocities are higher, which allows for the uninterrupted operation of the generator 24/7. In practice the nature of the wind velocity changing in time tends to include the features of both courses, and the mean energy additionally depends on the deterministic components: circadian and annual changes and long term trends.

At the current technological level of energy storage production, solving the problems related to the first type of breaks seems hard and economically not justified. Nevertheless, short breaks in the operation of wind sources can be effectively compensated with energy from appropriately selected energy storage, resulting in a partial stabilisation of the output parameters of a wind power plant connected to the grid, and also contribute to improving the quality of the generated electrical energy [15, 20, 21].

## 2. Cooperation of Energy Storage with Wind Turbine

### 2.1. Introduction

With regard to the breaks in the wind turbine operation caused by the stochastic nature of the wind velocity (energy) changes in time, it is necessary to find some engineering solutions preventing the related power cuts to the grid. Considering significant technical difficulty related to eliminating long-lasting power cuts, the methods allowing for preventing power cuts with maximum duration *T*_MAX_ can be considered sufficient but the parameter value is usually determined for a range up to several minutes.

One of the practically feasible methods is maintaining the power supplied to the electrical energy system at the assumed level with the value of *P*_3MIN_ in the reference periods. In order to maintain high quality of energy and stable operation of the system in the connection spot, the change in the power level supplied to the system from the value related to the wind turbine power curve to the value of *P*_3MIN_ should be as smooth as possible. It is also recommended to implement measures aimed at partial stabilisation of the system output power at the level of *P*_3MIN_ in periods with reduced wind energy. Such situations occur when the wind velocity value is *v*_*P*_3MIN__′ > *v*_*w*_ ≥ *v*_cut-in_, where *v*_*P*_3MIN__′ is the velocity corresponding to the power *P*_3MIN_ + *P*_PW_ (*P*_PW_ is the house load power of the analysed system). A complete stabilisation of the output power of the WT-ESS for all periods of the turbine operation at a reduced power requires using complex engineering systems and is economically not justified (very high investment expenditure).

The paper assumes that the implementation of the presented measure requires the use of energy storage with appropriate parameters and of appropriate type. The basic aim is to compensate the reduced power supplied by the wind turbine generator in the assumed periods with duration up to *T*_MAX_. The solution is of particular importance for geographical areas with the values of the average wind velocity not much higher than the cut-in velocity *v*_cut-in_ of the applied type of turbine. Appropriately selected turbine and energy storage leads to a creation of wind turbine-energy storage (WT-ESS) of a new quality, connected to the power grid, whose features on the one hand result from its being a renewable source of energy but on the other hand are similar to the characteristics of conventional sources [20, 22, 23].

### 2.2. Characteristics of Energy Storage Systems, Selecting the Energy Storage Type, Advantages and Disadvantages of Kinetic Storage

Accumulation of energy is a topical and economically expensive problem, of high technological complexity [11, 12]. The studies carried out in this field result, among others, from aspiration to improve energetic safety and from the need of long-term accumulation of very large amount of energy. The difficulties in accumulation of electric energy cause the indirect methods are most commonly used. Consequently, it reduces the efficiency of the process. Among the systems that make most often use of the above-mentioned method there are accumulator batteries (lead-acid and lithium-ion batteries), kinetic storage (flywheels), supercapacitors, superconducting magnetic energy storage (SMES), and compressed air energy storage (CAES) [4, 11, 12, 24].

In case of the storage designed for operation in renewable energy systems the requirements related to their energetic capacity, rated power, charging rate, and durability, and the range of operating temperature results from specific conditions of wind turbine and photovoltaic panel operation, caused directly by weather conditions. The changes in temperature, humidity, pressure, and so forth not only directly affect the equipment but also contribute to stochastic changes of input values delivered by the aforesaid types of the sources.

It was assumed for purposes of the research that functionality of the energy storage in electric power grids is described with the use of a set of parameters including: power and energy densities (W/L and Wh/L, resp.)—determining possible recovery of usable current (power) and energetic capacity, durability (the number of charging-discharging cycles), depth of discharge, the range of operating temperature, discharge rate and transition rate between the operating states, efficiency, unit cost of the equipment, converted to power or unit energy (cost/kW, cost/kWh) and physical dimension of the system.  Table 1 presents a comparison of the most important usable parameters of the above mentioned energy storage types [10, 12].

In order to carry into effect the algorithm proposed in the paper and aimed at partial stabilization of the power delivered to the system from a wind source a storage is necessary which renders possible a so-called short-term accumulation of energy. It is designed for equalizing the output power of the system in time intervals below 1 h (usually 0,25 h). Such systems are required to deliver the energy to the electric power grid immediately after activation of the storage (with very short delay) and to maintain it at the rated power level in the assumed time [10]. Taking into account a single wind turbine, an energy storage cooperating with it should have the average energetic capacity (usually from tens to hundreds kWh), high charging rate (comparable to discharging rate—in the range of minutes), rated power in the range from tens to hundreds kW, very short time of transition between charging and discharging stages (below 1 s) and the range of operating temperature corresponding to yearly temperature variations characteristic for the definite geographic location. Moreover, the storage should be composed of modules allowing for simple development of the system [25].

The SMES storage must be excluded from cooperation with wind turbines due to their low energy density (0,5 Wh/L ÷ 10 Wh/L). Usable current value of a single module reaches even several kA (the superconducting technology and significant reduction of active power loss), nevertheless, the time of cooperation with the system is too short as compared to the one required according to the assumption. Similarly, the CAES storage is excluded too, due to the need of building large systems (pressure vessels) or using a precisely imposed location of the system (natural reservoirs, e.g., old mine excavations, etc.), and relatively poor efficiency of the system. Such a type of the storage is characterized by too long deployment time (from several to 10 minutes) as compared to real dynamics of wind energy variations. On the other hand, high power density is an advantage of this storage type. Nevertheless, in case of the time of energy recovery below one hour this advantage is not decisive for the choice of the storage type [10]. From the group of considered solutions of the problem the supercapacitors must be removed too. This is caused by very low energy density (2 Wh/L ÷ 10 Wh/L) which precludes gaining proper capacity and maintaining the output power at required level within the time from ten to twenty minutes.

Hence, the most important types of energy storage feasible for practical application of the proposed method of equalizing the output power of a wind turbine with stochastic character of the input function are secondary electrochemical cells—accumulators and flywheels [10, 12].

Among the advantages of the flywheels as compared to electrochemical cells (lead-acid and lithium-ion batteries) there are constant value of energetic capacity in the whole range of operational temperature (−35°C to +40°C), covering yearly variations of weather conditions, very high number of charging and discharging cycles reaching millions (lifetime 15–20 years) and short duration of storage charging (approximating the discharging time with rated power) [10, 12, 16]. Two first features allow to locate the storage in direct proximity of the turbine and to operate it without any restrictions within the turbine lifetime (15–20 years). High charging rate [16] enables to use the wind energy even in case of quick variations, without the need of using faster energy storage devices as energetic buffers. Additionally, the kinetic storage is characterized by high efficiency (from 80% to 95%), remarkably higher as compared to lead-acid batteries (75%–80%). For the recent solutions their efficiency is higher even than the one of lithium-ion batteries (83%–86%). It should be noticed that the system occupies relatively small space—a group of modules may be often closed in a container ready for transportation to another location [10, 12].

One of the features of the kinetic storage, that might be considered as a fault as compared to accumulator battery is lower energy density (in case of lead-acid battery from 50 Wh/L to 100 Wh/L, while for the lithium-ion one—from 200 Wh/L to 350 Wh/L). Another fault of them is due to high degree of self-discharge (several percent per hour). Nevertheless, the above-mentioned features are not decisive for cooperation between the wind turbine-energy storage system and the electric power grid, since the storage is not required to be characterized by very large energetic capacity and the storage charging and discharging processes last below 1 hour—usually no more than twenty minutes. The investment cost of flywheels, converted to unit power or unit energetic capacity, is several times higher than that of the lead-acid or lithium-ion batteries. Hence, economical aspects of the use of such systems must be considered as their fault worsens appraisal of the technology of kinetic storage [10, 12].

Obtaining high energy values requires a high flywheel velocity, which entails the use of modern composite materials. Their density is several times lower than the density of steel, and the boundary strength *σ*_max⁡_ related to the presence of high radiation forces is much higher, which results in obtaining the value of characteristic energy several times higher (W/kg). Detailed information on this matter is presented in the paper [26]. Low idle changes and a relatively high total system performance (usually of ca. 86%) are mainly achieved by using magnetic bearings and the rotor operation in a vacuum with the pressure values of about 10^−3^ bar [7].

Based on the comparison of technical parameters of the above-mentioned types of energy storage and considering the economic aspects (periodical replacement of batteries) a flywheel type of energy storage was assumed for cooperation with the wind turbine [9].

### 2.3. Algorithm of a Flywheel Energy Storage Cooperation with a Wind Turbine (Farm)

**Figure 3:**
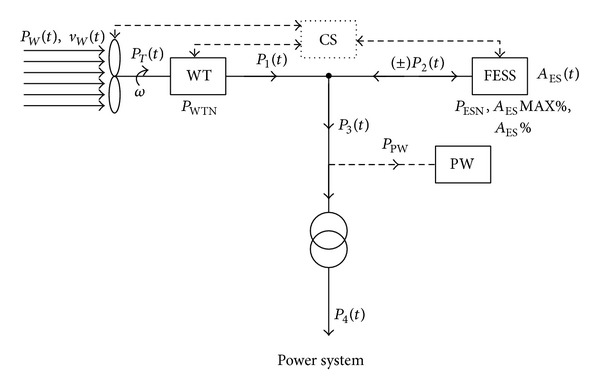
Construction diagram, principles of operation and power flow in the WT-FESS (WT—wind turbine, FESS—flywheel energy storage, CS—control system, *P*_*T*_(*t*)—mechanical power, *P*_WTN_—wind turbine nominal power, and *P*_PW_—the system house load power).

According to the established assumptions, a wind turbine with the nominal power *P*_WTN_ and specific power curve *P*_1_ = *f*(*v*_*w*_) working with flywheel energy storage form a complex power system (WT-FESS). Its basic goal is to deliver a relevant level of active power to the power grid system also in the periods when the wind velocity *v*_*w*_ is below *v*_cut-in_. The basic diagram of a flywheel-electrical system is presented in Figure 3. The kinetic energy of wind is transformed in the turbine wheel into the shaft (or gear) and generator rotary motion. According to the turbine power curve, active power *P*_1_(*t*) is obtained at the system outlet. The storage operates with the active output power *P*_2_(*t*) variable in time; the power can be positive (energy released to the power grid—unloading), negative (energy taken from the generator—loading) or zero energy (idle state of complete unloading of the storage). Hence the storage energy *A*_ES_(*t*) also varies in time and its value ranges from zero to the nominal capacity *A*_ES*N*_. The current energy value tends to be expressed in the percentage of nominal value, with the use of factor *A*_ES%_(*t*).

Active power *P*_3_(*t*), which is an algebraic sum of momentary powers *P*_1_(*t*) and *P*_2_(*t*) with deducted house load power *P*_PW_, is released to the system. Due to an automatic change in the WT-FESS configuration, its value also varies in time *P*_PW_ = *f*(*t*). According to the assumptions given in Section 2.1 while releasing energy from the storage to the grid, the minimum output power value *P*_3MIN_ is obtained. However, it covers periods of time with a specific duration (maximum duration *T*_MAX_) and depends on meeting several conditions given further on in the algorithm.

The system presented in Figure 3, depending on the momentary value of the wind velocity *v*_*w*_(*t*) and the energy storage loading *A*_ES_(*t*), can be in one of the four characteristic states:

 (i) autonomic operation of the turbine generator (*v*_*w*_(*t*) > *v*_*P*_3MIN__′ and *A*_ES%_(*t*) ≥ *A*_ES%MIN_) or (*v*_*P*_3MIN__′ > *v*_*w*_(*t*) ≥ *v*_cut-in_ and  *A*_ES%_(*t*) = 0):
  (2a) $$\begin{array}{c} P_{3}\left(t\right)=P_{1}\left(t\right)-P_{\text{PW}}\left(t\right), \end{array}$$
  where *A*_ES%MIN_ is the minimum level of the storage energy not resulting in its supplementary loading under favourable wind conditions,
 (ii) generator operation with supplementary loading of the energy storage (*v*_*w*_(*t*) > *v*_*P*_3MIN__′, *A*_ES%_(*t*) < *A*_ES%MIN_):
  (2b) $$\begin{array}{c} P_{3}\left(t\right)=P_{1}\left(t\right)-P_{2}\left(t\right)-P_{\text{PW}}\left(t\right), \end{array}$$
 (iii) simultaneous operation of the generator and energy storage (*v*_*P*_3MIN__′ > *v*_*w*_(*t*) ≥ *v*_cut-in_, *A*_ES%_(*t*) > 0):
  (2c) $$\begin{array}{c} P_{3}\left(t\right)=P_{1}\left(t\right)+P_{2}\left(t\right)-P_{\text{PW}}\left(t\right), \end{array}$$
 (iv) autonomic operation of the energy storage (*v*_*w*_(*t*) < *v*_cut-in_, *A*_ES%_(*t*) > 0):
  (2d) $$\begin{array}{c} P_{3}\left(t\right)=P_{2}\left(t\right)-P_{\text{PW}}\left(t\right). \end{array}$$

The transition between the above-mentioned states is a continuous and dynamic process, depending on the stochastically changing atmospheric conditions and the current and previous system arrangement. A single continuous operating period of energy collecting from flywheel energy storage is limited with the *T*_MAX_ algorithm parameter.

## 3. Selecting the Energy Storage Volume for Working with a Wind Turbine

### 3.1. Statistical Energy Analysis of the Course of Wind Velocity Changes *v*_*w*_ = *f*(*t*)

Based on theoretical analysis and the conducted tests it was determined that the measurement courses of the wind velocity changes *v*_*w*_ = *f*(*t*) can be used for identifying the minimum capacity of the flywheel energy storage *A*_ESMIN_ that will meet the assumptions of the algorithm of WT-FESS cooperation with the power grid system, according to Section 2.3. It was established that the knowledge of the output parameters of the WT-FESS (time *T*_MAX_, power *P*_3MIN_) and technical parameters of the turbine (nominal power *P*_WTN_, cut-in velocity *v*_cut-in_, power curve *P*_1_ = *f*(*v*_*w*_)) and of the energy storage (idle losses Δ*A*_ES*j*%_, performance at loading *η*_*F*+_ and unloading *η*_*F*−_, nominal power *P*_ES*N*_, continuous maximum power *P*_ESMAX_) are additionally required.

**Figure 4:**
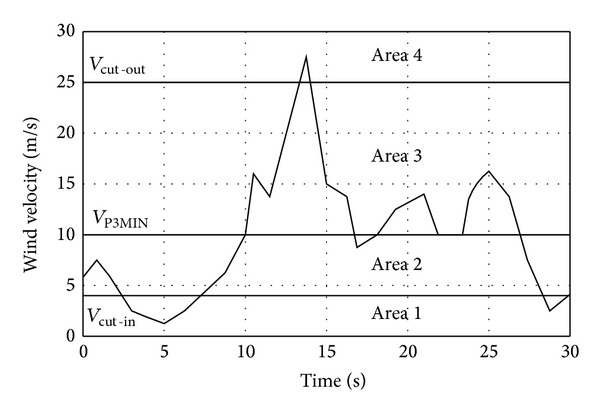
Course of wind velocity changes *v*_*w*_ = *f*(*t*) with marked areas used for determining the value of statistical and energetic parameters of WT-FESS.

Assuming the above-mentioned principle of the WT-FESS operation, on a sample course of the wind velocity changes (Figure 4) horizontal lines identifying the parameters characteristic of the system are marked: the turbine cut-in velocity *v*_cut-in_, velocity *v*_*P*_3MIN__ of obtaining the power *P*_3MIN_ + *P*_PW_ and the turbine cut-out velocity *v*_cut-out_ were marked. This way the course *v*_*w*_ = *f*(*t*) is divided into four areas, where a set of statistical and energy parameters characterising the WT-FESS in the specific geographical location can be determined.

In the area 1 the wind velocity meets the requirement *v*_*w*_(*t*) < *v*_cut-in_, and the generator power is *P*_1_ = 0. In practice such periods can last from several seconds to many days. In order to identify the required capacity of a flywheel energy storage *A*_ESMIN_ information about subsequent breaks of the specific type and their average duration is necessary. The parameters proposed and used in further analysis for the area include the average *T*_1AVG_ and maximum *T*_1MAX_ duration of power generation breaks (stochastic wind velocity changes) not exceeding the set value of the factor *T*_MAX_, *k*_1_ series coefficient determining the average number of subsequent breaks separated with one turbine operation interval at power guaranteeing the energy storage loading (*P*_1_ > *P*_3MIN_ + *P*_PW_) and the summary turbine operation time in the area *T*_1WT_ for the assumed period of analysis *T*_*a*_.

Area 2 covers the wind velocity range meeting the requirement *v*_cut-in_ < *v*_*w*_ ≤ *v*_*P*_3MIN__′. Information concerning the average* m T*_2AVG_ and maximum *T*_2MAX_ duration of intervals not exceeding the set value of *T*_MAX_, the average generator power *P*_1AVG2_ and the total turbine operating time in the area *T*_2WT_ for the assumed period of analysis *T*_*a*_ is determined in the area.

The system operation in area 3 (wind velocity *v*_*w*_ ≥ *v*_*P*_3MIN__′) allows for controlled loading of the storage according to its current energy status *A*_ES_(*t*). The average generator power *P*_1AVG3_ and the total turbine operating time in the area *T*_3WT_ for the assumed period of analysis *T*_*a*_ is determined for the area.

Area 4 covers the turbine cut-out periods due to excess wind velocity *v*_*w*_ ≥ *v*_cut-out_, which can additionally cause mechanical damage. Moreover, the following values of electrical energy generated by the reference type of turbine are determined for the total period *T*_*a*_ and areas 2 and 3: *A*_WT_, *A*_2WT_, and *A*_3WT_, respectively.

According to the description above, sets of measurement points whose values constitute the average wind velocity from the period Δ*t*_*m*_ and the duration of 48 seconds are analysed. Hence 1800 measurement points are recorded within 24 hours, and their number amounts to 657 thousand within one year. For high power wind turbines (hundreds kW and more) the moments of inertia of rotating elements are so high that the quoted measurement period is sufficient for the goals presented in the paper. All measurements used in the paper were made with a rotating anemometer placed at 10 m above the land level.

From the point of view of the analysed subject matter it is important to compare the values and relationships between the suggested statistical energy parameters for two characteristic periods of a calendar year: autumn-winter and spring-summer. For many geographical locations, including the South Eastern Europe, the autumn-winter period has greater wind energy that the spring-summer one and the differences can be of several dozen percent. Another important element covers determining the impact of the change in the WT-FESS input and output parameters, in particular in the parameter of time *T*_MAX_ and power *P*_3MIN_ on the proposed statistical and energetic factors at the established course of wind velocity changes and the type of the employed wind turbine.

Tables 2(a), 2(b), 3(a), and 3(b) present a comparison of the results of a statistical-energetic analysis of the course of wind velocity changes *v*_*w*_ = *f*(*t*) recorded for three periods in 2010: period I (autumn-winter 1 January 2010–31 March 2010), period II (spring-summer: 1 June 2010–31 August 2010) and period III (1 January 2010–31 December 2010) at the assumed time *T*_MAX_ = 600 seconds and two powers at the WT-FESS outlet *P*_3MIN_ = 200 kW (Tables 2(a) and 2(b)) and *P*_3MIN_ = 300 kW (Tables 3(a) and 3(b) in periods with reduced wind energy (*v*_*w*_(*t*) < *v*_cut-in_ and *v*_*P*_3MIN__′ > *v*_*w*_(*t*) ≥ *v*_cut-in_). The analysis was made for Enercon E53 turbine with nominal power 800 kW, at recalculating the wind velocity value to the rotor hub centre (*h*_*w*_ = 60 m) according to the relationship (1).

### 3.2. Identifying the Boundary Capacity *A*_ESMIN_ of a Flywheel Energy Storage

The WT-FESS operation according to the assumptions of the algorithm presented in Section 2.3. requires using a flywheel energy storage with appropriate capacity. The author's research on the analysis of the measurement courses of the wind velocity changes *v*_*w*_ = *f*(*t*) for a period of several years for one geographical location lead to determining an empirical relationship identifying the value of the minimum storage capacity *A*_ESMIN_ that guarantees correct operation of the analysed system. The relationship includes technical parameters of the storage and wind turbine and statistical energy parameters of the measurement courses of the wind velocity changes defined in Section 3.1.

The presented relationship consists of segments corresponding to the turbine operation areas separated in Figure 3. A corrective segment related to the storage additional loading conditions and its ability to use the excess energy generated by the turbine (*P*_1_ > *P*_3MIN_) was also taken into account. Considering these elements in determining the minimum capacity *A*_ESMIN_ of a storage intended for working with a selected type of wind power plant in a specific geographical location, the following relationship was proposed:

(3) AESMIN=k1ηES−·T1AVGg·P3MIN +k1ηES−·k2·T2AVGg·(P3MIN−P1AVG2d) +PESN·kESj%100·TjAVGg   −k3·k4·ηES+T3AVGd·(P1AVG3−P3MIN),

where *T*_1AVG_^*g*^, *T*_2AVG_^*g*^, *T*_3AVG_^*d*^ is the upper (*g* index) and lower (*d* index) confidence limit for the subsequent mean time values *T*_1AVG_, *T*_2AVG_, and *T*_3AVG_ (Tables 2(a), 2(b), 3(a), and 3(b)), *k*_ES*j*%_ is the idle losses of the flywheel storage expressed in percent of its nominal power *P*_ES*N*_, *T*_*j*AVG_^*g*^ is the upper confidence limit of the storage operation on idle gear (the value stands for the mean time between subsequent periods of the storage energy use in areas 1 and 2 whose duration does not exceed the maximum natural unloading time storage *T*_ESR*j*_), *η*_ME+_, *η*_ME−_are the flywheel energy storage performance in the loading and unloading process, *k*_2_ is the correction factor (*k*_2_ = 0 for *P*_3MIN_ ≤ *P*_2AVG_^*d*^ and *k*_2_ = 1 for *P*_3MIN_ > *P*_2AVG_^*d*^), *k*_3_ is the coefficient of the storage additional loading conditions:

(4) $$\begin{array}{c} k_{3}=\frac{P_{\text{WTN}}-P_{4\text{MIN}}}{P_{\text{WTN}}-P_{1\text{MIN}}}, \end{array}$$

identifying the turbine power margin that can be used during the storage additional loading, where *P*_1MIN_ stands for the minimum turbine power value corresponding with the wind velocity *v*_cut-in_, *k*_4_ is the ability to use excess power:

(5) $$\begin{array}{c} k_{4}=\{\begin{array}{cc} 1 & \text{for}\;\;P_{3\text{AVG}}-P_{3\text{MIN}}\leq P_{\text{ES}N},\\ \frac{P_{\text{ES}N}}{P_{3\text{AVG}}-P_{3\text{MIN}}} & \text{for}\;\;P_{3\text{AVG}}-P_{3\text{MIN}}>P_{\text{ES}N}. \end{array} \end{array}$$

The other factors and parameters used in the relationship (3) are described in the previous section of the paper.

The first three components of the relationship (3) help determine partial capacities related to stabilisation of a power plant output power for areas 1 and 2 at the established maximum continuous duration of the turbine operation with reduced power (*P*_1_ < *P*_3MIN_) and idle loses of the flywheel energy storage Δ*P*_ES*j*_ (*P*_2_ = 0, *A*_ES_(*t*) > 0). The last element is of corrective nature and in special cases reduces the value of the identified capacity. Additionally, it happens that the real capacity of the storage *A*_ESMIN_ must not be lower than the *A*_ESMIN_ determined from the relationship (3) and in practice depends on the nominal data of the modules available for the selected storage type and the possibility of their combining.

### 3.3. Changes in the Capacity *A*_ESMIN_ in the Function of WT-FESS Parameters

A computational application was developed with the use of the analysis algorithm of the measurement courses of wind velocity changes *v*_*w*_ = *f*(*t*) proposed in Section 3.1 and empirical relation (3), in the  .NET environment (language C#). With regard to a large number of measurement points covering the period of one year and the related long times of statistical analysis, the Task Parallel Library was used, for parallel execution on multicore system which allowed to significantly reduce the total time of calculations.

With the use of the developed application, families of characteristics *A*_ESMIN_ = *f*(*T*_MAX_) and *k*_1_ = *f*(*T*_MAX_) were determined for the established set of power values *P*_3MIN_ and particular geographical location. Based on them it is possible to evaluate the behaviour of the WT-FESS when wind turbines with identical nominal power are used, to differentiate the mounting height of the wind wheel and to analyse the system for different periods of the same year and to compare several years. The above-mentioned families of characteristics were determined separately for two periods of the same year: autumn-winter and spring-summer. The conducted calculations used the values of standard deviations and confidence ranges, assuming the confidence factor of 0,95, which were determined for statistical and power parameters presented in Tables 2(a), 2(b), 3(a), and 3(b).

**Figure 5:**
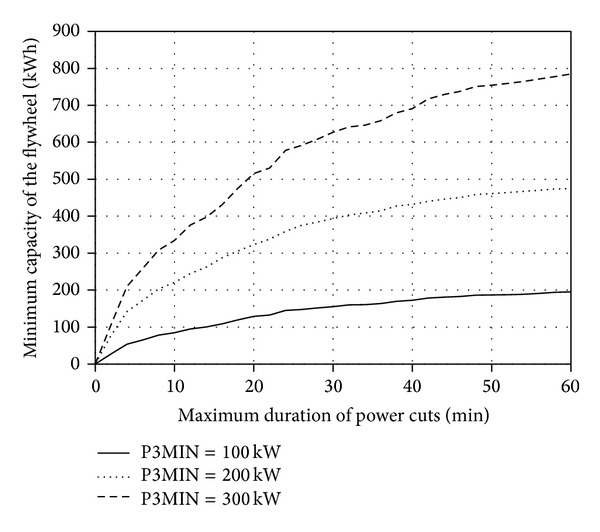
Family of characteristics *A*_ESMIN_ = *f*(*T*_MAX_) for Enercon E53 turbine, height: *h*_*w*_ = 60 m for the period between 1 Jan. 2010 and 31 Mar. 2010.

**Figure 6:**
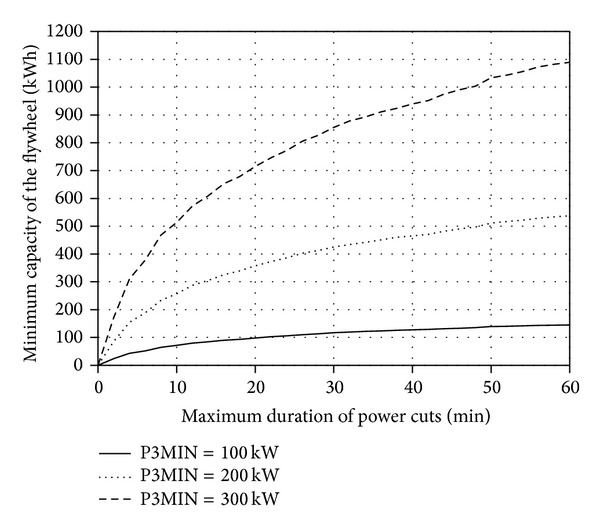
Family of characteristics *A*_ESMIN_ = *f*(*T*_MAX_) for Enercon E53 turbine, height: *h*_*w*_ = 60 m for the period between 1 June 2010 and 31 Aug. 2010.

**Figure 7:**
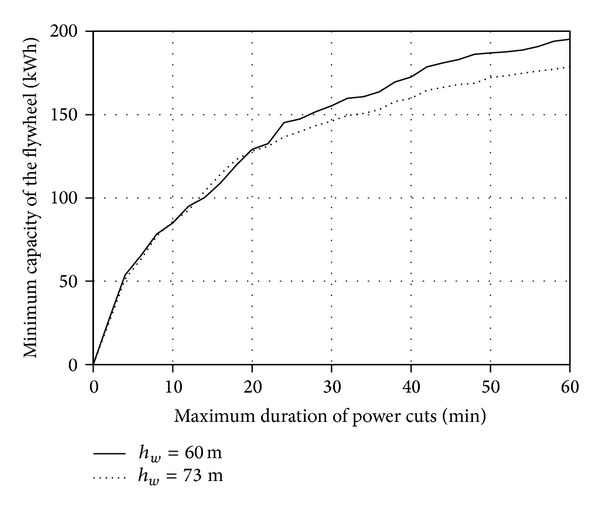
Family of characteristics *A*_ESMIN_ = *f*(*T*_MAX_) for Enercon E53 turbine and the system power of *P*_3MIN_ 100 kW for the period between 1 Jan. 2010 and 31 Mar. 2010.

**Figure 8:**
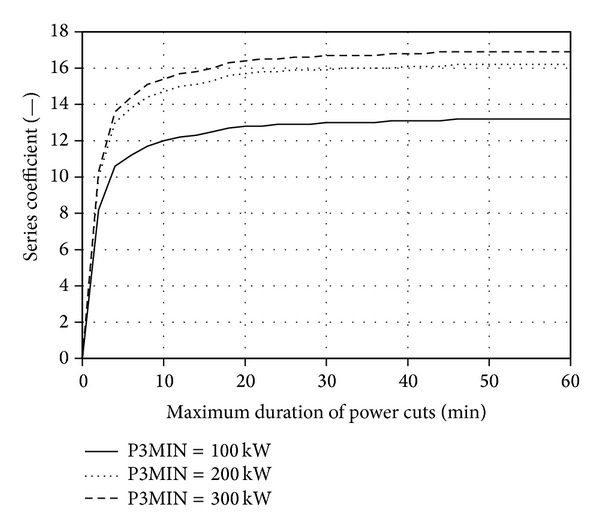
Family of characteristics *k*_1_ = *f*(*T*_MAX_) for Enercon E53 turbine, height: *h*_*w*_ = 60 m for the period between 1 Jan. 2010 and 31 Mar. 2010.

Figures 5, 6, 7, and 8 present the discussed families of characteristics determined for two periods: from 1 January 2010 to 31 March 2010 and from 1 June 2010 to 31 August 2010, assuming the mounting height of Enercon E53 wind turbine converter of *h*_*w*_ = 60 m and *h*_*w*_ = 73 m and three power values of the WT-FESS *P*_3MIN_ = 100 kW, 200 kW and 300 kW.

**Figure 9:**
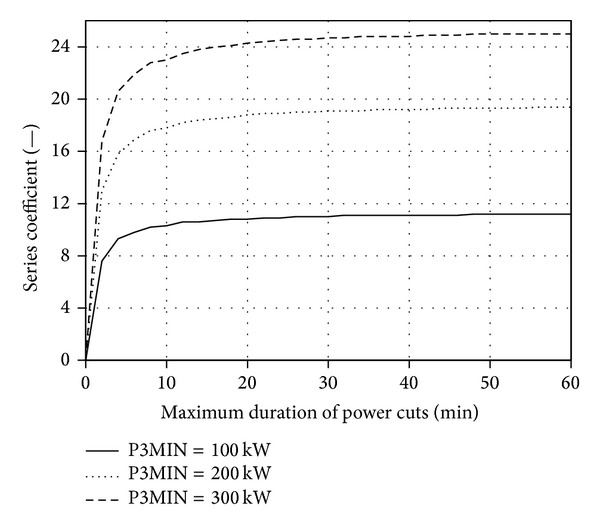
Family of characteristics *k*_1_ = *f*(*T*_MAX_) for Enercon E53 turbine, height: *h*_*w*_ = 60 m for the period between 1 June 2010 and 31 Aug. 2010.

**Figure 10:**
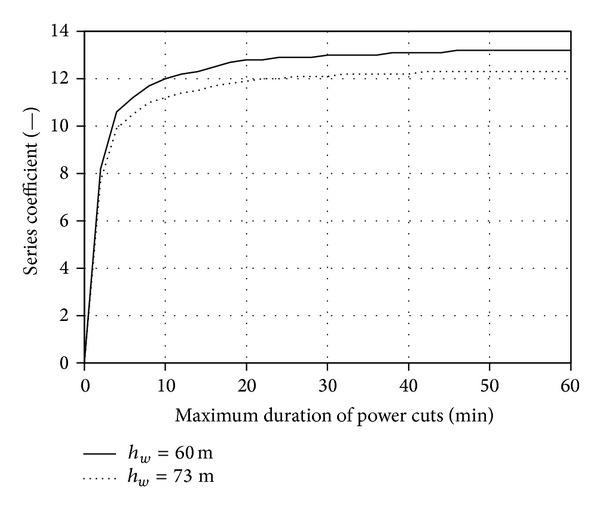
Family of characteristics *k*_1_ = *f*(*T*_MAX_) for Enercon E53 turbine and the system power of *P*_3MIN_ 100 kW for the period between 1 June 2010 and 31 Aug. 2010.

Additionally, the investigation covered the impact of the change in the wind converter mounting height on the above-mentioned characteristics. Two mounting heights of the E53 turbine converter quoted in the catalogue were employed while implementing the task (*h*_*w*_ = 60 m and *h*_*w*_ = 73 m) alongside with a method of calculating the wind velocity against the measurement height, according to the relationship (1). Figures 9 and 10 present the results of calculating the changes in *A*_ESMIN_ capacity and *k*_1_ multiplication factor for the system power *P*_3MIN_ = 100 kW, for the period between 1 January 2010 and 31 March 2010.

Extending the maximum acceptable time *T*_MAX_ of the turbine operation with a limited or zero power (*P*_1_ < *P*_3MIN_) results in an increase in the flywheel energy storage *A*_ESMIN_ allowing for the WT-FESS operation according to the proposed algorithm—Section 2.3. The change is non-linear, and reveals the greatest dynamics at lower time values *T*_MAX_. It mainly results from the nature of the changes in the multiplication factor *k*_1_ (Figures 7 and 8). The differences in the characteristics curves *k*_1_ = *f*(*T*_MAX_) between the spring-summer and autumn-winter period result from different average wind velocity and the dynamics of the wind velocity changes in time. Analysing the obtained characteristics one can note their similarities within the dynamics of the *A*_ESMIN_ storage capacity changes for both analysed periods. The determined capacity *A*_ESMIN_ for the spring-summer period is higher than for the autumn-winter period, which is mainly caused by higher average values of the wind velocity (kinetic energy) in the winter period. Lower values of the multiplication factor for the winter period can be attributed to higher dynamics of the wind velocity change *v*_*w*_ in time and the change in the speed of switching between the turbine operating areas marked in Figure 3.

## 4. Simulation of WT-FESS Operation under Conditions of Stochastic Wind Energy Change

### 4.1. Simulator Model

Verification of the proposed algorithm of wind turbine cooperation with a flywheel energy storage (WT-FESS) required developing an analytical and numerical model and implementing a simulator of the analysed system operation. With regard to the necessary application of proprietary computational methods covering, statistical analysis of the wind change velocity measurement data, identifying the minimum capacity of a flywheel energy storage, and analysing the changes in the storage energy in time, it is reasonable to develop our own simulation application. The set goals include:

 (i) verifying the effectiveness of the proposed method of determining the minimum capacity of a flywheel energy storage *A*_ESMIN_ intended for working with a wind turbine, at the established geographical location,
 (ii) carrying out tests of the system behaviour under simulation and real conditions of the wind energy changes in time,
 (iii) analysing the results of WT-FESS operation as compared to the independent operation of the wind turbine under constant wind conditions.

It was assumed that the correctness of determining the minimum capacity of a flywheel energy storage *A*_ESMIN_ intended for working with a wind turbine is established based on the value of a percentage factor of eliminating the acceptable cut-outs *k*_*L*%_. It is the relationship between the summary working time of a generator with power below *P*_3MIN_ in unit periods and duration not exceeding *T*_MAX_, compensated with the flywheel storage energy, and the summary time of all periods of the generator operating at a power not exceeding *P*_3MIN_ and duration not exceeding *T*_MAX_ (including not compensated periods), in the assumed period of analysis *T*_*a*_, expressed in percent.

A set of *N* wind velocity values discrete in time is the simulator input obtained by measurements. According to Section 3.1. of the paper, each measurement point makes the average wind velocity for the period Δ*t*_*m*_ 48 seconds long.

In the numerical algorithm of the simulator, regardless of the energy storage operation state, one should consider idle losses related to mechanical resistance in the system, feeding of magnetic bearings and maintaining the specific vacuum level in the rotating mass housing. If the energy storage is in an idle state they are taken into account as *k*_ES*j*%_ factor. At loading and unloading the idle losses are included in the process efficiency, whereby the efficiency was assumed as identical in both cases and its value is *η*_ES_.

The momentary power of a wind turbine generator *P*_1_(*t*) is determined with the use of the energy curve stored in a discrete form in the database. The values of the generator power are determined for each of the established points *N* separating the time periods Δ*t*_*m*(*i*)_ for *i* = 1,2,…, *N* − 1. For the initial *t*_*ms*(*i*)_ and final *t*_*me*(*i*)_ time of the Δ*t*_*m*(*i*)_ period, wind velocities amounting to *v*_*ws*(*i*)_ and *v*_*we*(*i*)_ respectively and the generator power *P*_1*s*(*i*)_ and *P*_1*e*(*i*)_ related to them are determined. The average turbine power in the range Δ*t*_*m*(*i*)_′ and value *P*_1AVG(*i*)_ is used for the calculations made in the WT-FESS operation simulator. The changes in the energy storage power *P*_2_(*t*) are established based on the relationships from (2a) to (2d), whereas the output power *P*_3_(*t*) of the system is identified based on the determined values of *P*_1_(*t*) and *P*_2_(*t*) and the house load power *P*_PW_(*t*).

The energy state of the storage in discrete moments of time *t*_*k*_ for *k* = 0,1, 2,…, *N* is determined based on the initial storage loading condition (for *k* = 0, *A*_ES*N*_ ≥ *A*_ES0_ ≥ 0), previous changes in the storage *P*_2_(*t*) and turbine *P*_1_(*t*) power, its efficiency, and coefficient of idle losses. The value of energy for discrete time *t*_*k*_ (*t*_*k*_ = *k* · Δ*t*_*m*_) is determined by adding (considering the sign) the energy gains in all time ranges Δ*t*_*m*_ preceding the *t*_*k*_ point. The storage energy in the moment of time *t*_*k*_ can, thus, be expressed as

(6)   AES(tk=k·Δtm)=AES0+∑i=1k(b(i)·ηES·P2(i)·Δtm) −∑i=1k−1(c(i)·1ηES·P2(i)·Δtm) −∑i=1k(d(i)·kESj%·PESN·Δtm100),

where *i* is the time step index, *k* is the final time step index used according to the relationship *t*_*k*_ = *k* · Δ*t*_*m*_, to determine the time *t*_*k*_, *P*_ES*N*_ is the nominal power of energy storage, *P*_2(*i*)_ is the established value of the energy storage loading or unloading power as the average value for the initial and final point of the time range Δ*t*_*m*_, *b*_*i*_, *c*_*i*_, *d*_*i*_ ∈ {0,1} are the coefficients from sets *b*, *c*, and *d*, respectively identifying the storage state for the time periods (loading, unloading, idle).

For numerical implementation of proposed model  .NET platform, MS Visual C# language and ADO.NET technology for handling the relational database of the wind turbines parameters were used. Elements of object-oriented software were applied for building the programme structures. A library of classes intended for representing the structure and operating principle of the following WT-FESS elements: wind turbine, flywheel energy storage, control system, method of selecting *A*_ESMIN_ storage capacity and identifying the storage energy state at any moment of time *t*_*k*_ were developed. In relation to a very time-consuming nature of the calculations covering a statistical energy analysis of the discrete course of wind velocity changes in time, elements of calculation paralleling were used. That is why Task class was used to divide the calculations onto logical cores of the processor intended for PCs and workstations.

### 4.2. Results of Simulation Analyses

Simulation tests of a WT-FESS working with the power grid system were carried out for two types of inputs test input *v*_WT_ = *f*(*t*) and real input *v*_*w*_ = *f*(*t*). Two configurations of the system with different nominal power *P*_ES*N*_, limit capacities *A*_ESMIN_ and initial loading states *A*_ES0%_ of the storage (option I and II—Table 4) were used for the tests. The real input case is covered by parameters presented in Table 4 as option III. ENERCON E 53 turbine with the power of *P*_WTN_ = 810 kW and established generation characteristics was used in all tests.

**Figure 11:**
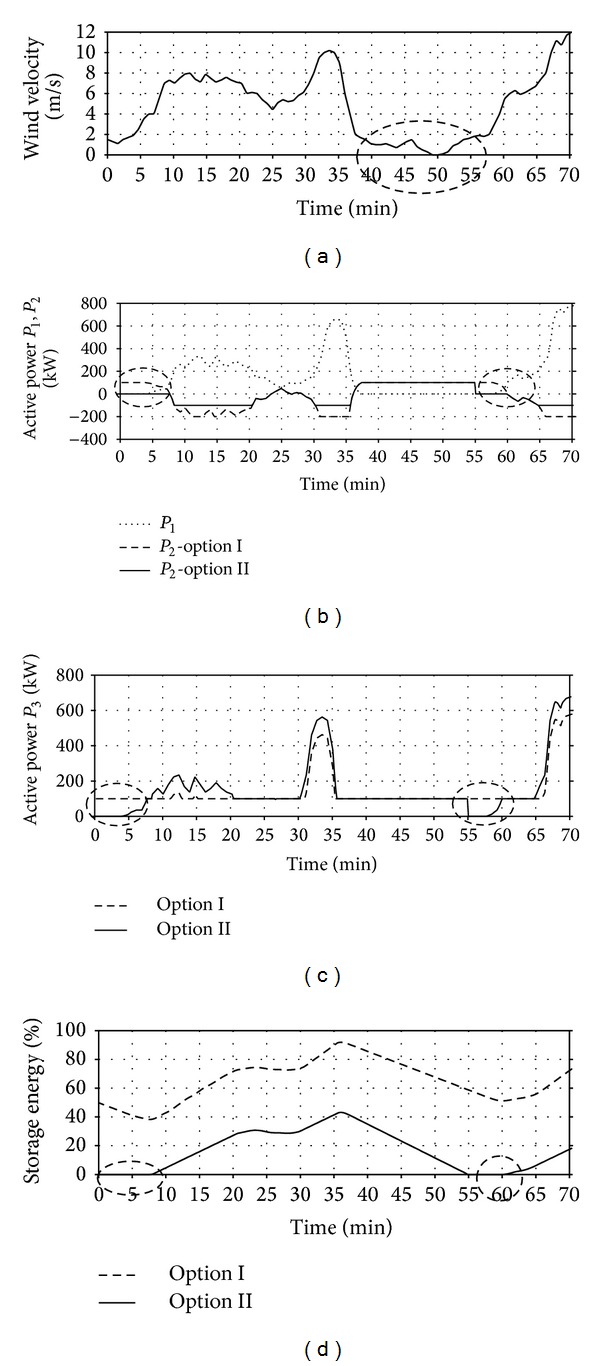
Courses of changes in WT-FESS parameters obtained in the developed simulator for the test input *v*_WT_ = *f*(*t*) and options I and II of calculations (Table 4): (a) wind velocity *v*_WT_, (b) power *P*_1_ and *P*_2_, (c) power *P*_3_, (d) storage loading state *A*_ES%_.

The first part of the tests was done for the input *v*_WT_ = *f*(*t*), whose curve is presented in Figure 11(a). The analysis covers changes in the wind velocity during 70 minutes, including fluctuations from the cut-in velocity *v*_cut-in_, to the velocity *v*_*N*_ when the turbine reached the nominal power *P*_WTN_. The velocity changes *v*_WT_ in time were selected so that in the assumed period of analysis *T*_*a*_ the system WT-FESS reached all working states defined in the defined algorithm (Section 2.3) and shifted between them at diversified dynamics.

The other part of the tests covered a simulation of the investigated system operation for a real input in a form of the curve of wind velocity changes from the one indicated in the geographical location reference for the period between 3 March and 6 March 2008. The nominal (limit) capacity *A*_ESMIN_ of the storage used for the tests was determined for an identical location but using measurement data for the spring-summer period in 2010.

According to the assumptions presented in Section 2.3., the numerical simulator model covers four operating states of the system depending on the wind energy, system parameters, and current and previous values of the energy storage. The results of the performed simulations were presented in a form of power curves of the generator *P*_1_(*t*), storage *P*_2_(*t*) (considering the sign) and the output power of the system *P*_3_(*t*) and a relative percent storage loading *A*_ES%_(*t*) for the assumed period of analysis *T*_*a*_.

Figure 11 shows the results of WT-FESS operation simulation conducted for the test input and two parameter options of the tested system (Table 4). With regard to the short period under analysis and the related high readability in Figures 11(b)–11(d), the curves for the aforementioned parameters are presented simultaneously for two simulation options (Table 4).

As a result of the wind velocity drop below *v*_cut-in_ in the period between 37 and 57 minutes, if the turbine works independently, it is disconnected from the power grid system (Figure 11(a)—circled with an intermittent line). However, considering the turbine cooperation with the storage, the break was eliminated thanks to the previously stored energy (Figures 11(b) and 11(c)). For option II, considering the assumption of zero storage energy at the beginning of the analysis period (*A*_ES0_ = 0), the stored energy was not sufficient to eliminate the entire break, which resulted in the turbine cut-out after 20 minutes. A similar situation occurred in the first period of the system operation (to ca. minute 4). The enumerated periods are circled with an intermittent line in Figures 11(c) and 11(d). It is the evidence of too low capacity of the applied energy storage, resulting from extremely difficult storage operating conditions not included in the confidence ranges of statistical energy parameters used in the relationship (3).

**Figure 12:**
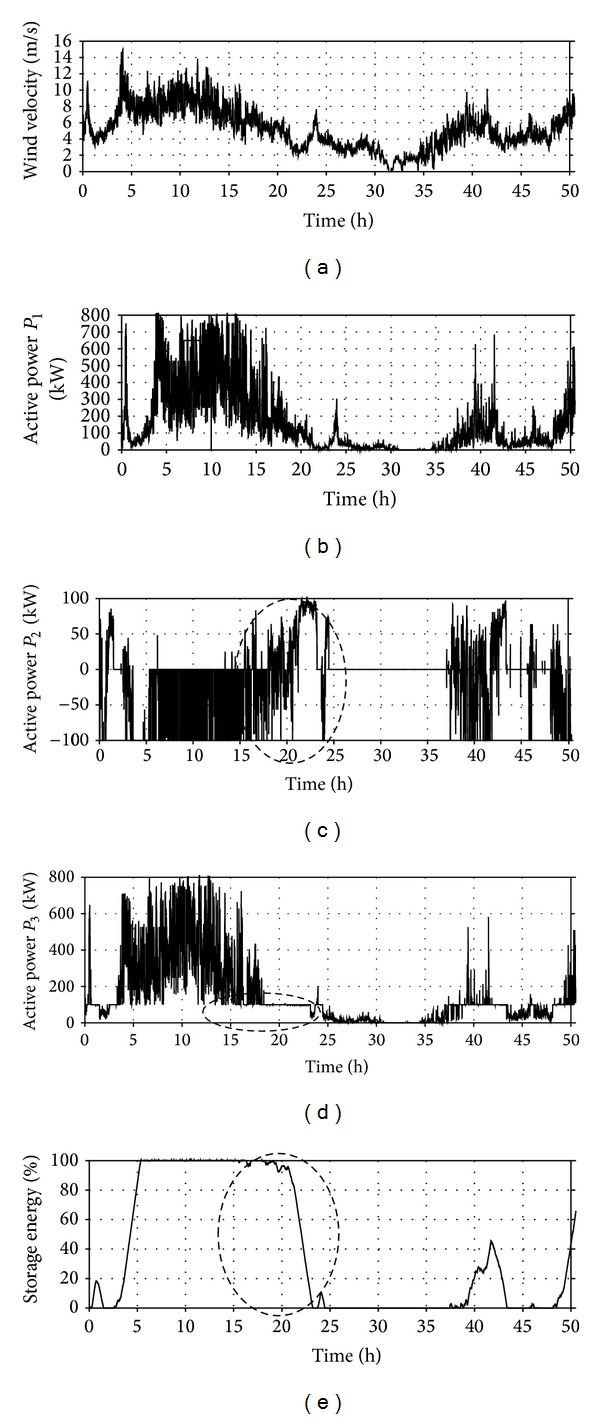
Courses of changes in WT-FESS parameters obtained in the developed simulator for the test input *v*_WT_ = *f*(*t*) for the period between 3 March–6 March 2008 (calculation option III): (a) wind velocity *v*_*w*_, (b) power *P*_1_ (c) power *P*_2_, and *P*_3_ (d) storage loading state *A*_ES%_.

Figure 12 shows the curves of some selected simulator parameters for WT-FESS operation at real input (option III—Table 4).

The analysis of the system operation for a real input covers 50 hours from the period between 3 March 2008 and 6 March 2008, with diversified wind conditions (Figure 12(a)). Next to high wind energy periods (e.g., between the system operation hour 5 and 20) there are periods with boundary energy values from the point of view of the assumed WT-FESS operation parameters (e.g., between hour 20 and 30). This type of periods accumulates breaks in the turbine operation, which are short according to the definition presented in Section 1 of the paper and should be additionally compensated with energy stored in the storage. Furthermore, a period of long-lasting decrease in the wind velocity below the cut-in velocity (between system operation hour 31 and 34) can be additionally seen in Figure 12, whose impact on the system operation will not be analysed in detail.

From the point of view of the developed algorithm, the most important periods are the ones with boundary (limit) values of the wind velocity (energy). The implemented algorithm of WT-FESS cooperation with the power grid system assumes stabilisation of the output power *P*_3_ of the system at the assumed level *P*_3MIN_, besides eliminating short breaks. It applies to periods where the wind velocity allows for reaching the turbine power *P*_3MIN_ > *P*_1_ > 0 (area 2 in Figure 4) and the assumed duration up to *T*_MAX_. In the analysed period *T*_*a*_ the greatest number of wind velocity changes corresponding to the transition between areas 1 and 2 (Figure 4) occurs between hour 15 and 25 of the system operation. This period is circled with an intermittent line in Figures 12(c)–12(e). Unloading of the storage energy is used for eliminating breaks in the turbine operation (*P*_1_ = 0) and equalising the system output power *P*_3_ with the value of *P*_3MIN_ (Table 4, option III) assumed in the algorithm. It is also loaded between the storage unloading periods (positive power *P*_2_), when the power values *P*_2_ are negative (Figure 12(c)).

## 5. Comments and Conclusions

Operation of wind sources in geographical locations with moderate wind conditions may generate a number of problems related to their cooperation with the power grid system. The basic reason for such occurrence is stochastically changing kinetic energy of the wind and construction characteristics of the turbines. One of the solutions to mitigate the effect of frequent cut-outs of such sources from the grid is using energy storage. Implementing the proposed algorithm of the wind turbine can control the system operation—flywheel energy storage system cooperation with the grid that allows for eliminating a large number of short breaks using the previously stored energy. The author proposed an algorithm using the features of flywheel energy storage, mainly the short period of their loading and shifting between the loading and unloading state, as well as low dependence of the real capacity on temperature. Equalising the active power released to the power grid system at the assumed level *P*_3MIN_ is done for the breaks in the turbine operation and periods when the turbine reaches the power *P*_1_ < *P*_3MIN_ at maximum duration *T*_MAX_. The results obtained by simulation (Figures 11 and 12) are the evidence of good efficiency of the developed algorithm and improving the conditions of the wind turbine cooperation with the power grid system. The number of the turbine cut-outs from the grid at appropriately selected flywheel energy storage capacity decreases significantly, which results in an improved quality of electrical energy and the source stability.

Correct operation of the above-mentioned system requires determining the minimum (boundary) capacity *A*_ESMIN_ of the applied energy storage. The process can be conducted in different ways but the author of the paper suggests a proprietary concept based on statistical energy analysis of the measurement time series of changes in the wind velocity in the analysed geographical location for a period of at least one year (Tables 2(a), 2(b), 3(a), and 3(b)). The minimum capacity of the storage *A*_ESMIN_ required for the assumed algorithm at maintaining the specified parameters of cooperation with the power grid system is established based on the empirical relationship (3) connecting the energy storage and wind turbine parameters and states, as well as the results of statistical energy analysis of the measurement curves *v*_*w*_(*t*). Seasonality of the average wind energy demonstrated based on the tests (Tables 2(a), 2(b), 3(a), and 3(b)) indicated the need to consider this fact in determining the limit storage capacity *A*_ESMIN_. The simulation results confirm that if this fact is accounted for while establishing the value of *A*_ESMIN_ the real percent index of eliminating the acceptable breaks (duration up to *T*_MAX_) is between 75% and 85%. Not meeting this condition results in a significant decrease in the process of eliminating short breaks in the wind turbine operation defined in the paper.

In the author's opinion the statistical energy parameters proposed and determined for the measurement curves can be compared and taken into account while designing WT-FESS systems in various geographical locations. Based on the values of the parameters presented in Tables 2(a), 2(b), 3(a), and 3(b) one can draw more detailed conclusions on the nature of wind conditions in the examined location (energy, dynamics of changes, etc.), similarly to the wind conditions class according to IEC 61400-1. As a result of implementing heuristic methods it is additionally possible to select the optimum components of the WT-FESS (turbine type, tower height, type and size of storage) as regards the unit cost of electrical energy generation.

It was established based on the conducted statistical energy analyses of the curves *v*_*w*_ = *f*(*t*) (Tables 2(a), 2(b), 3(a), and 3(b)) and the tests according to the implemented method of determining the capacity *A*_ESMIN_ that for a specific geographical location, conclusions concerning mutual relations between the parameters characterising the WT-FESS and cooperation with the power grid can be formulated. With this in mind, a series of calculations was made whose results are presented as curves *A*_ESMIN_ = *f*(*T*_MAX_) at *P*_3MIN_ = const (Figures 4 and 5), and *A*_ESMIN_ = *f*(*T*_MAX_) at *h*_*w*_ = const (Figure 6). The coefficient of series *k*_1_ has a major impact on the capacity value *A*_ESMIN_ and the shape of the enumerated characteristics. Considering the dependence of the coefficient *k*_1_ on the turbine construction, wind conditions and the assumed value *P*_3MIN_ calculations were made and characteristics determined for *k*_1_ = *f*(*T*_MAX_) at *P*_3MIN_ = const (Figures 8 and 9) and *k*_1_ = *f*(*T*_MAX_) at *h*_*w*_ = const (Figure 10).

The families of the aforementioned curves are typical of a particular geographical location, the parameters of the system elements (*P*_WTN_, *P*_ESN_, *h*_TW_) and its cooperation with the power grid (*T*_MAX_, *P*_3MIN_). They can be used for an approximate determination of the minimum (limit) capacity of the storage *A*_ESMIN_ when different values of the wind wheel mounting height, power change *P*_3MIN_ and time of the eliminated breaks *T*_MAX_ are used.

The choice of energy accumulation system in the form of flywheels is an effective solution that enables to fulfill the assumptions formulated for the algorithm of WT-FESS system cooperation with the electric power grid. Exchange of the storage for accumulator batteries would worsen the system properties because of long charging time (the lead-acid batteries), capacity variations (particularly in winter) and shorter lifetime (in higher temperature). On the other hand, the use of supercapacitors would result in significant growth of the cost, since they should be distinguished by high electric capacity. Hence, it appears that despite the disadvantages mentioned in Section 2.2 the kinetic energy storage complies with the largest number of required qualities. Moreover, development of the technology allows forecasting reduction of the kinetic storage prices in the future and their more common use, particularly in the field of renewable power engineering.

The results presented in the paper are a basis for further research particularly in two basic spheres. The first of them consists in analysis of operation simulation of a WT-FESS system within one year, with consideration of repeated changes in wind power. The other includes optimization of the WT-FESS system aimed at definition of such structure of the system for which the unit cost of electric power production is possibly the lowest for the considered geographic location.

## Figures and Tables

**Table 1:** Specification of the most important usable parameters of selected types of the energy storage [10, 12].

Energy storage type	Round-trip efficiency [%]	Energy density [Wh/l]	Power density [kW/l]	Cycle life/calendar life	Depth of discharge [%]	Self-discharge [%]	Deployment time	Charging time	Operating temperature [°C]
Flywheel	80–95	20–200	up to 10	Many millions/15 years–20 years	75	2–5/h	10 ms	Minutes	−35 ÷ +40
Supercapacitor	90–94	2–10	up to 15	Up to one million/15 years	75	Very slow	<10 ms	Seconds	−40 ÷ +65
Lithium-ion battery	83–86	200–350	0,1–3,5	/5–20 years (according to temperature)	100	5/monthly	3 ms–5 ms	Hours	−20 ÷ +50
Lead-acid battery	75–80	50–100	0,01–0,5	500–2000 cycles/5–15 years (according to temperature)	70	0,1–0,4/daily	3 ms–5 ms	Many hours	0 ÷ 40
CAES	60–70	3–6	n.a.	Unlimited/25 years	35–50	0,5–1/daily	3 min–10 min	Hours	−30 ÷ 60
SMES	80–90	0,5–10	1–4	Unlimited/20 years	100	10–15/daily	1 ms–10 ms	Seconds-minutes	n.a.

**(a):** 

Period	*T*_*a*_	*A*_WT_		*A*_2WT_		*A*_3WT_		*T*_1AVG_ [s]	*T*_2AVG_ [s]	*k*_1_ [—]
	[Days]	[MWh]	[/%]	[MWh]	[%]	[MWh]	[%]			
1 Jan. 2010–31 Mar. 2010	90	397,0	100	59,2	14,9	337,8	85,1	114,1	136,0	14,7
1 June 2010–31 Aug. 2010	92	256,4	100	89,8	35,0	166,7	65,0	119,3	143,8	17,9
1 Jan. 2010–31 Dec. 2010	365	1501,6	100	314,7	21,0	1186,8	79,0	121,4	138,9	17,6

**(b):** 

Period	*T*_WT_		*T*_1WT_		*T*_2WT_		*T*_3WT_		*P*_1AVG2_ [kW]	*P*_1AVG3_ [kW]	*P*_1AVG_ [kW]
	[h]	[%]	[h]	[%]	[h]	[%]	[h]	[%]			
1 Jan. 2010–31 Mar. 2010	2160	100	585,2	27,1	916,1	42,4	658,7	30,5	62,4	514,0	251,4
1 June 2010–31 Aug. 2010	2208	100	221,8	10,0	1559,1	70,6	427,1	19,3	55,4	398,4	129,2
1 Jan. 2010–31 Dec. 2010	8760	100	1197,9	13,7	5083,4	58,0	2478,7	28,3	59,6	482,3	197,9

**(a):** 

Period	*T*_*a*_	*A*_WT_		*A*_2WT_		*A*_3WT_		*T*_1AVG_ [s]	*T*_2AVG_ [s]	*k*_1_ [—]
	[Days]	[MWh]	[/%]	[MWh]	[%]	[MWh]	[%]			
1 Jan. 2010–31 Mar. 2010	90	397,0	100	98,2	24,7	298,8	75,3	114,1	137,4	15,4
1 June 2010–31 Aug. 2010	92	256,4	100	131,3	51,2	125,2	48,8	119,3	146,5	23,0
1 Jan. 2010–31 Dec. 2010	365	1501,6	100	487,9	32,5	1013,6	67,5	121,4	141,5	20,8

**(b):** 

Period	*T*_WT_		*T*_1WT_		*T*_2WT_		*T*_3WT_		*P*_1AVG2_ [kW]	*P*_1AVG3_ [kW]	*P*_1AVG_ [kW]
	[h]	[%]	[h]	[%]	[h]	[%]	[h]	[%]			
1 Jan. 2010–31 Mar. 2010	2160	100	585,2	27,1	1075,1	49,8	499,7	23,1	89,3	602,0	251,4
1 June 2010–31 Aug. 2010	2208	100	221,8	10,0	1729,7	78,3	256,5	11,6	73,7	503,2	129,2
1 Jan. 2010–31 Dec. 2010	876	100	1197,9	13,7	5789,9	66,1	1772,2	20,2	81,8	579,1	197,9

The calculations use the power curve and other E53 turbine parameters presented in the manufacturer's technical catalogue [19].

**Table 4:** List of technical parameters of WT-FESS used in simulation tests.

Option	*P*_ESN_ [kW]	*A*_ES0%_ [%]	*A*_ESMIN_ [kWh]	*T*_MAX_ [s]	*P*_3MIN_ [kW]	*k*_*j*%_ [%]	*P*_PW%_ [%]
I	200	50	100	1800	100	2	0.5
II	100	0	75	1800	100	2	0.5
III	100	0	150	600	100	2	0.5
